# Supplementary material for: Phosphatidylethanolamine Deficiency and Triglyceride Overload in Perilesional Cortex Contribute to Non-Goal-Directed Hyperactivity after Traumatic Brain Injury in Mice
Source: Biomedicines. 2022 Apr 15;10(4):914. doi: 10.3390/biomedicines10040914 (PMC9033131; doi:10.3390/biomedicines10040914)
Supplement: Supplementary file 1 [file biomedicines-10-00914-s001.zip › biomedicines-1663337-supplementary.pdf]

Supplementary Figures and Legends

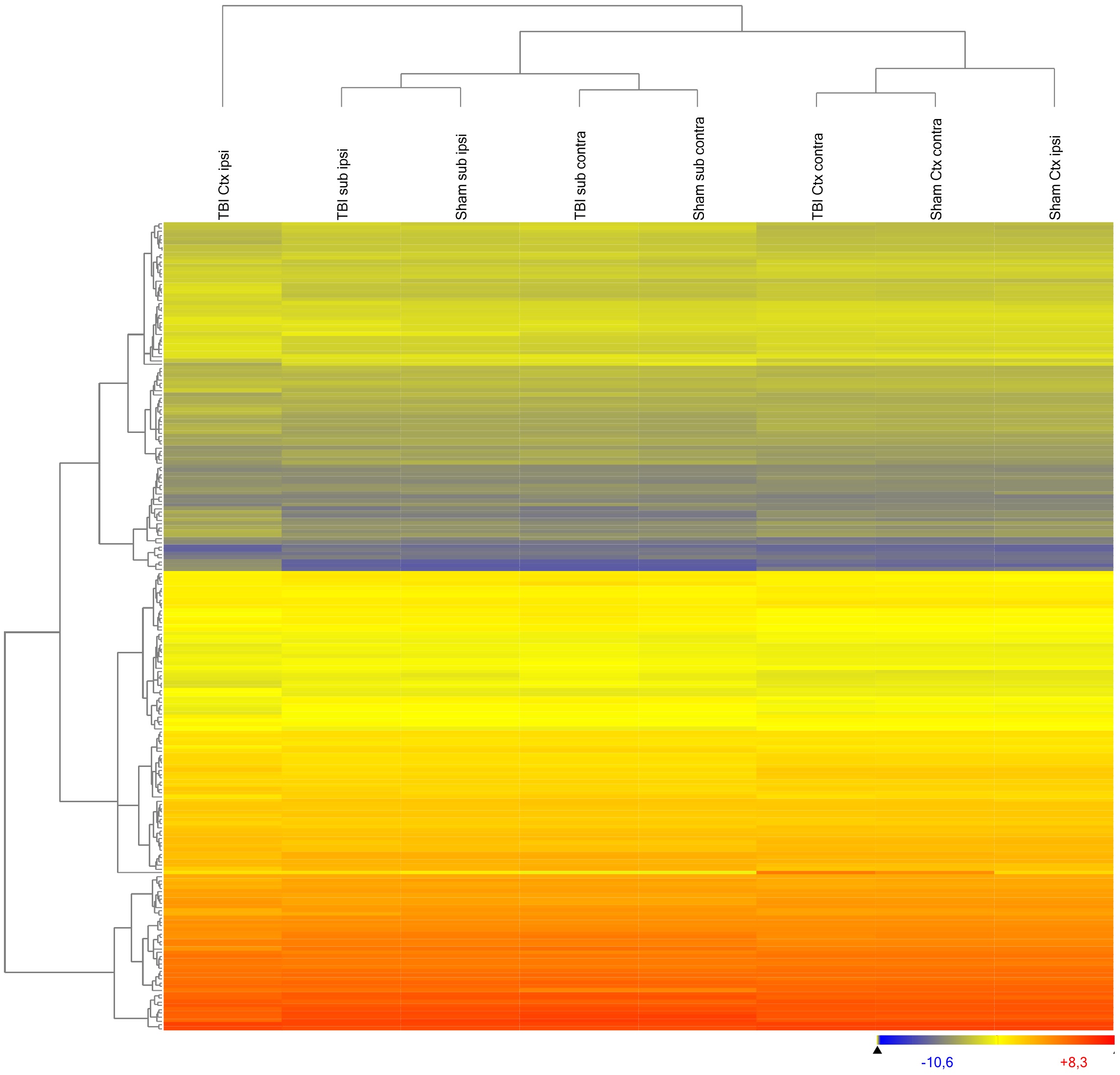

Supplementary Figure S1. Heatmap of the lipidome in ipsilateral and contralateral cortical and subcortical brain 5.6 months after TBI versus sham surgery. Each horizontal line is a lipid species. Lipids and mice were clustered using hierarchical clustering with Euclidean distance metrics. Mouse clusters are depicted as top dendrograms. Each group color column shows the mean of n = 15 TBI and n = 16 sham mice. The colour scale ranges from −3.5 to +3.5 SD.

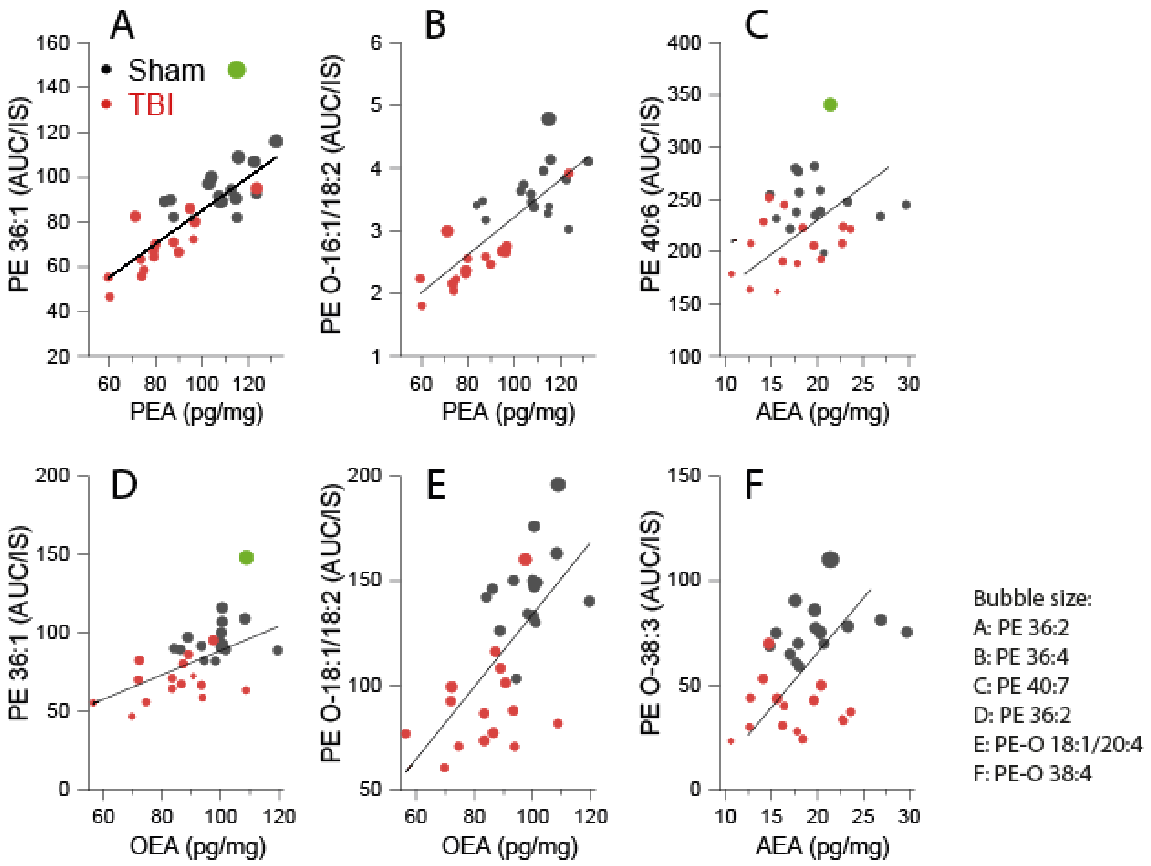

Supplementary Figure S2. Association of phosphatidylethanolamine species with endocannabinoids, palmitoylethanolamide (PEA), oleoylethanolamide (OEA) and anandamide (AEA). PE species were selected according to chain length to fit to the endocannabinoid (PEA 16C, OEA 18C, AEA 20C) or multiples of this chain length. The line shows the linear regression line, the shaded area is the 90% confidence interval. The slopes differed significantly from zero. AUC/IS: Ratio of the area under the curve of the analyte and the AUC of the internal standard. Bubble sizes show related lipid species. The green dots reveal outliers which were excluded from regression analyses. **A:** PEA versus PE 36:1; **B:** PEA versus PE O-16:1/18:2; **C:** PEA versus PE 40:6; **D:** PEA versus PE 36:1; **E:** PEA versus PE O 18:1/18:2; **F:** PEA versus PE O-38:3.

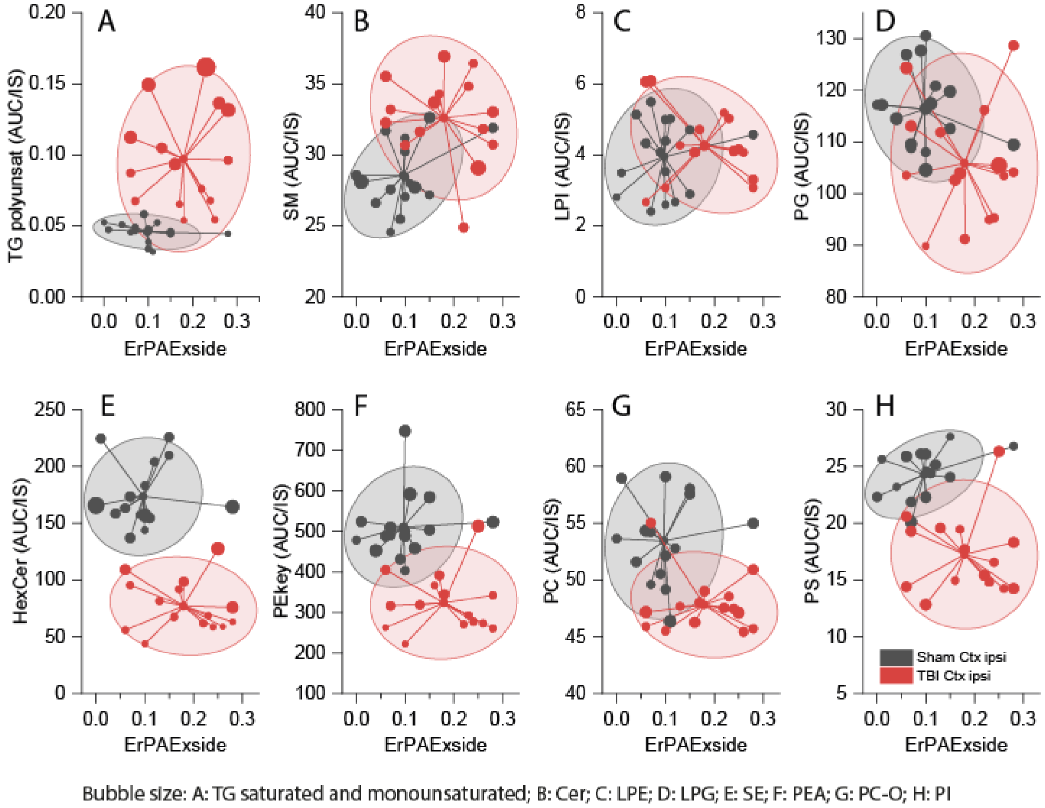

Supplementary Figure S3. Multi-panel associations of the proportion of side errors in the extinction phase of avoidance learning (ErrPAEx side) (X-axis) with lipid classes (Y-axis) in the ipsilateral perilesional cortex of sham (*n* = 16, black) versus TBI (n = 15, red) mice. High proportion of errors (ErrPAEx side) (X-axis) reveals rapid loss of memory. Daily proportions of errors were averaged over the observation time of the PAEx task (5 days), and the mouse's average was used for associations with lipid classes on the Y-axis. The bubble size is the respective lyso-form or a related lipid. AUC/IS (unit) is the ratio of the area under the curve of the analyte and the AUC of the internal standard. Individual lipid species of different chain lengths and saturation were summed per lipid class. Scatters represent individual mice. The ellipses show 85% CI of the prediction. **A**: PAEx errors versus polyunsaturated triacylglycerols (TG). Bubble size saturated and monounsaturated TG. **B**: PAEx errors versus sphingomyelins (SM). Bubble size ceramides. **C**: PAEx errors versus lysophosphatidylinositol (LPI). Bubble size LPE. **D**: PAEx errors versus phosphatidylglycerol (PG). Bubble size LPG. **E**: PAEx errors versus hexosylceramides. Bubble size sterol ester (SE). **F**: PAEx errors versus phosphatidylethanolamines (PE). Bubble size palmitoylethanolamide (PEA). **G**: PAEx errors versus phosphatidylcholines (PC). Bubble size PC-O. **H**: PAEx errors versus phosphatidylserine (PS). Bubble size phosphatidylinositol (PI).

Supplementary Table S1. Abbreviations of behavioral parameters of IntelliCage experiments.

IntelliCage Parameter	Explanation
**Visits**	Visits / h
**NPVisits**	Visits with Nosepoke without Licks / h
**LVisits**	Visits with Licks / h
**SVisits**	Visits without Licks and without Nosepokes / h
**NPVdur**	Median duration of Visits with NP w/out Lick (s)
**Nosepokes (NP)**	Mean number of Nosepokes during Visits with NP w/out Licks
**Licks**	Median number of Licks per Visit
**Lduration**	Median duration of Licking during a Visit (s)
**Sidedness**	Ratio of visits with first left versus first right NP of visits with NPs
**Mesor**	**M**idline **e**stimating **s**tatistic **o**f **r**hythm. The mesor is a circadian rhythm-adjusted mean based on the parameters of a cosine function fitted to the raw data of corner visits.
**Amplitude**	Difference between Mesor and Peak activity
**Acrophase**	Time to maximum activity after Light Off (Light off set to 0)

Supplementary Methods: Analytical materials

LC-HRMS lipidomic analyses materials

Water, isopropyl alcohol, methanol, acetonitrile (LC-MS grade) and methyl-tert-butyl-ether (MTBE, HPLC-grade) were purchased from Carl Roth (Karlsruhe, Germany). Ammonium formate (for mass spectrometry, ≥ 99.0 %) and ethanol (for GC, ≥ 99.8 %) were obtained from Sigma-Aldrich (Munich, Germany) and formic acid (98-100 %) from AppliChem (Darmstadt, Germany).

Internal standards and concentrations in working solution

**Analyte**	**Concentration (µg/ml)**
Arachidonic acid-d8	0.1
CE 18:1-d7	5
Cer d18:1/16:0-d7	0.02
Cholesterol-d7	1.5
DG 15:0/18:1-d7	0.3
LacCer d18:1/17:0	0.06
LPC 18:1-d7	0.3
LPC O-16:0-d4	0.02
LPE 18:1-d7	0.02
LPG 17:1	0.02
LPI 17:1	0.02
PC 15:0/18:1-d7	2
PC O-18:0/18:1-d9	0.2
PE 15:0/18:1-d7	0.1
PE O-18:0/18:1-d9	0.1
PG 15:0/18:1-d7	0.1
PI 15:0/18:1-d7	0.1
PS 15:0/18:1-d7	0.025
SM d18:1/18:1-d9	0.4
TG 15:0/18:1-d7/15:0	0.6

All IS were from Avanti Polar Lipids, Alabaster, AL, USA.

Instrumentation

Mass spectrometer	Orbitrap Exploris 480 (Thermo Fisher Scientific, Dreiech, Germany)<br>HESI-source
HPLC	Vanquish Horizon (Thermo Fisher Scientific, Dreiech, Germany)<br>Vanquish Compartment H<br>Vanquish Split Sampler HT<br>Vanquish Pump H
LC-column	Zorbax RRHD Eclipse Plus C8 1.8 µm 50 x 2.1 mm ID (Agilent, Waldbronn, Germany), heated at 40 °C, flow rate 300 µL/min<br>Precolumn: ZORBAX Eclipse Plus C8, 2.1 mm, 1.8 µm (Agilent, Waldbronn, Germany)
Solvent A	Water + 0.1% formic acid + 10 mM ammonium formate
Solvent B	Acetonitrile:isopropanole 2:3 (v/v) + 0.1% formic acid
Injection volume	2 µl (positive ion mode), 5 µl (negative ion mode)

LC-gradients of solvent A and solvent B

Time (min)	Sol. A (%)	Sol. B (%)
0.00	75.0	25.0
0.30	75.0	25.0
1.50	20.0	80.0
11.00	0.0	100.0
12.00	0.0	100.0
12.50	75.0	25.0
14.00	75.0	25.0
